# The Molecular Epidemiological and Immunological Characteristics of HIV-1 CRF01_AE/B Recombinants in Nanjing, China

**DOI:** 10.3389/fmicb.2022.936502

**Published:** 2022-07-15

**Authors:** You Ge, Yangyang Liu, Gengfeng Fu, Jing Lu, Xiaoshan Li, Guoping Du, Gaoqiang Fei, Zemin Wang, Han Li, Wei Li, Pingmin Wei

**Affiliations:** ^1^Department of Epidemiology and Health Statistics, School of Public Health, Southeast University, Nanjing, China; ^2^Institute of HIV/AIDS/STI Prevention and Control, Jiangsu Provincial Center for Diseases Control and Prevention, Nanjing, China; ^3^Department of Lung Transplant Center, Nanjing Medical University Affiliated Wuxi People's Hospital, Wuxi, China; ^4^Department of Southeast University Hospital, Southeast University, Nanjing, China; ^5^Department of Quality Management, Children's Hospital of Nanjing Medical University, Nanjing, China

**Keywords:** HIV-1, CRF01_AE/B recombinants, Bayesian evolution analysis, transmission network, disease progression, immune reconstruction

## Abstract

Human immunodeficiency virus-type 1 (HIV-1) CRF01_AE/B recombinants are newly emerging strains that are spreading rapidly in Southern and Eastern China. This study aimed to elucidate the molecular epidemiological characteristics of HIV-1 CRF01_AE/B recombinants in Nanjing and to explore the impact of these novel strains on the immunological status. A total of 1,013 blood samples from newly diagnosed HIV-1-infected patients were collected in Nanjing from 2015 to 2019, among which 958 partial Pol sequences were sequenced successfully. We depicted the molecular epidemiological characteristics of CRF01_AE/B recombinants by the molecular evolutionary analysis, Bayesian system evolution analysis, and transmission network analysis. The generalized additive mixed model was applied to evaluate the CD4^+^ T-cell count change of CRF01_AE/B recombinants. The Kaplan–Meier analysis was performed to assess the time from combined antiretroviral therapy (cART) initiation to immune reconstruction. We have identified 102 CRF01_AE/B recombinants (102/958, 10.65%) in Nanjing, including CRF67_01B (45/102, 44.12%), CRF68_01B (35/102, 34.31%), and CRF55_01B (22/102, 12.57%). According to the Bayesian phylogenetic inference, CRF55_01B had a rapid decline stage during 2017–2019, while CRF67_01B and CRF68_01B have experienced a fast growth phase during 2014–2015 and then remained stable. We have constructed 83 transmission networks, in which three larger clusters were composed of CRF67_01B and CRF68_01B. CRF01_AE/B recombinants manifested a faster decrease rate of CD4^+^ T-cell count than CRF_07BC but similar to CRF01_AE. The probability of achieving immune reconstruction in CRF01_AE/B recombinants was lower than CRF07_BC in the subgroup of baseline CD4^+^ T-cell count at cART initiation <300 cells/μl. In summary, CRF67_01B and CRF68_01B were the major strains of CRF01_AE/B recombinants in Nanjing, which have formed large transmission clusters between Nanjing and other provinces. CRF01_AE/B recombinants might be associated with rapid disease progression and poor immune reconstruction. The continuous epidemiological monitoring of CRF01_AE/B recombinants should be highly emphasized.

## Introduction

The human immunodeficiency virus-type 1 (HIV-1) is characterized by extensive genetic variability due to high mutation and recombination rates of HIV genome. The HIV-1 virus can be divided into four groups according to genetic sequence differences in various genomic regions, namely, M, O, N, and P (Robertson et al., 2000). The HIV-1 M group that dominates the HIV pandemic consists of A–D, F–H, J, K, and recombinant forms (Robertson et al., 2000). Abundant recombinant forms are one of the hallmarks of HIV-1. The recombinant HIV-1 genomes that share the same recombination breakpoints between the same parents and have been identified in at least three individuals without epidemiological relationship were called circulating recombinant forms (CRFs) (Beamud et al., 2019). In China, CRF07_BC, CRF01_AE, CRF08_BC, and B sub-types have been the main circulating strains (Xiang et al., 2012; Li et al., 2016). Owing to the co-circulation of multiple lineages of HIV-1 strains, various novel CRFs have emerged in China, especially the strains that recombined from CRF01_AE and B subtypes (CRF01_AE/B recombinants). The CRF01_AE/B recombinants were the first CRF identified in this century in China (Gan et al., 2021). The prevalence of CRF01_AE/B recombinants has been increasing in Southern and Eastern China (Vrancken et al., 2020; Zhang et al., 2021). As one of the most developed provinces in Eastern China, various CRF01_AE/B recombinants have been found in Jiangsu Province (Guo et al., 2014; Han et al., 2015; Yin et al., 2019). Nanjing, the provincial capital city of Jiangsu Province, ranks in the top three of the cumulative number of reported HIV-infected patients in the whole province. Our previous study has indicated that CRF01_AE was the most prevalent strain in Nanjing (Li et al., 2019). However, a few studies have systematically investigated the molecular epidemiology features of CRF01_AE/B recombinants in Nanjing. It would be helpful for monitoring and precisely preventing these strains by understanding the transmission mode of CRF01_AE/B recombinants in-depth.

It has been widely recognized that HIV-1 subtypes may affect disease progression and immune recovery of patients (Taylor et al., 2008; Keller et al., 2009; Amornkul et al., 2013; Li et al., 2022). Several studies have revealed that patients infected with CRF01_AE showed an increased rate of CD4^+^ T-cell count decline (Ng et al., 2011; Cui et al., 2019), whereas CRF07_BC was associated with relatively slow HIV disease progression (Ye et al., 2022). The impaired immune recovery after the combined antiretroviral therapy (cART) has also been reported in patients infected with CRF01_AE cluster 1 (Li et al., 2022). As the newly emerging strains, the impact of CRF01_AE/B recombinants on disease progression and immune reconstruction efficacy has not been clarified yet.

In this study, we aimed to elucidate the molecular epidemiology characteristics of CRF01_AE/B recombinants in Nanjing from 2015 to 2019 and investigated the impact of CRF01_AE/B recombinants on disease progression and immune reconstruction by taking two of the most prevalent strains in Nanjing (i.e., CRF_01AE and CRF_07BC) as the control.

## Materials and Methods

### Study Subjects

Patients who had been newly diagnosed with HIV-1 infection within Nanjing's districts of Qinhuai, Xuanwu, Qixia, Jiangning, and Gulou have been enrolled in the study during 2015–2019. The inclusion criteria for samples selection were as follows: (1) HIV enzyme-linked immunosorbent assay was positive and confirmed *via* HIV-1 Western blot test as well; (2) newly diagnosed without cART; and (3) obtaining the participant's informed consent in oral or written forms. We collected 10 ml of peripheral blood from each patient using an ethylenediaminetetraacetic acid (EDTA) anticoagulation tube and separated the plasma within 12 h. According to the instructions of BD Multitest CD3/CD8/CD45/CD4 (FITC/PE/PerCP/APC), the CD4^+^ T-cell count of HIV-1-infected patients was measured by flow cytometry (FACSCalibur, Becton and Dickinson Company, USA). The plasma HIV-1 viral load was quantified by the AMPLICOR HIV-1 MONITOR TEST (Roche, Basle, Switzerland).

### HIV-1 Partial Pol Gene Amplification

As previous described (Li et al., 2020), viral RNA was extracted from 200 μl plasma samples and then reverse-transcribed to single-stranded cDNA by following the manufacturer's instructions. The extracted RNA was used for subsequent reverse-transcription polymerase chain reaction (RT-PCR), and nested PCR was carried out to generate the partial Pol region. The length of the HIV partial Pol region generated using nested PCR was 1,060 bp (HXB2: 2,253–3,312) including the complete protease (PR) and the first 300 codons of reverse-transcriptase (RT) genes. The DNA amplification product with correct position of the band identified by gel electrophoresis was sequenced.

### HIV-1 Sub-Typing

We examined the sequence quality by submitting all the assembled sequences to the HIV-1 sequence quality control tool (https://www.hiv.lanl.gov/content/sequence/QC/index.html). The recombinant strains were identified by the Recombination Identification Program (RIP; Siepel et al., 1995) and Jumping Profile HMM (jpHMM; Schultz et al., 2009) online tools in the HIV database. The Pol region sequence of each HIV-1 genotype was retrieved from the Los Alamos National Laboratory HIV-1 (LANL, https://www.hiv.lanl.gov/). The phylogenetic tree was constructed to determine the HIV-1 genotype using the FastTree software.

### Molecular Evolutionary Analyses of CRF01_AE/B Recombinants

We first searched the 10 sequences with the highest similarity to the partial Pol area of the Nanjing sequence using the BLAST tool (http://blast.ncbi.nlm.nih.gov/). The duplicate sequences and sequences with incomplete information (e.g., time and region) were deleted. The sequence alignment was then completed using the gene cutter online tool (https://www.hiv.lanl.gov/content/sequence/GENE_CUTTER/cutter.html). The Bio Edit software was applied to examine the alignment quality and perform the manual correction. Finally, we established the phylogenetic tree of CRF01_AE/B recombinants *via* the approximate maximum likelihood method (aML). We adopted generalized time reversible (GTR) + gamma (G) + invariable sites (I) as the nucleotide substitution model. The phylogenetic tree based on aML was visualized using the Figtree V1.4.4 software. The criteria for the epidemic branch are as follows: (1) number of Nanjing sequence in a cluster ≥20 and (2) bootstrap value ≥0.90 (de Oliveira et al., 2017).

### Bayesian Evolution Analysis of CRF01_AE/B Recombinants

To inferring the time to most recent common ancestor (tMRCA) of CRF01_AE/B recombinants from Nanjing, we operated the BEAST v1.10.4 software to perform the Bayesian evolution analysis (Chen et al., 2009). The optimal nucleotide substitution model was set as HKY + gamma. The Markov chain Monte Carlo (MCMC) analysis was run for 30,000,000 generations and sampled every 3,000 generations. According to the coefficient of variation index, the loose molecular clock model was selected to construct the Bayesian skyline plot (BSP) for inferring with the dynamic changes of the epidemic branches. We checked the effective sample size (ESS) using the Tracer v1.7.1 software (http://beast.community/tracer) and inspected whether the model to converge at the cutoff of ESS > 200. We removed 10% of the initial tree to generate the largest credible evolutionary branch tree using the Tree Annotator v1.10.4 software.

### Transmission Network Analysis

The molecular transmission network based on the partial Pol sequence of the Nanjing and reference sequence was constructed to infer the potential transmission relationship using the HyPhy software (Pond et al., 2005). The Tamura-Nei 93 (TN93) method was used to calculate the pairwise genetic distance. The threshold of transmission network was set at a pairwise genetic distance of ≤1.0%.

### Immunological Status Analysis of CRF01_AE/B Recombinants

With subtypes of CRF_01AE and CRF_07BC as controls, we investigated the impact of CRF01_AE/B recombinants on the change of the CD4^+^ T-cell count before cART and effectiveness of immune reconstruction after cART, respectively. The flowchart of the immunological status analysis is shown in Supplementary Figure 1.

In the part of evaluation of CD4^+^ T-cell count variation, the CD4^+^ T-cell count from the time of diagnosis to the cART initiation among cART-treated patients and the CD4^+^ T-cell count from the time of diagnosis to the last follow-up among cART-naïve patients has been collected. The time point of the last follow-up was 2021. All patients included had at least two CD4^+^ T-cell count records in different years. As described previously (Touloumi et al., 2013; Wei et al., 2021), we performed the square root transformation of the CD4^+^ T-cell count for linearizing the annual changes.

In the part of the assessment of the efficacy of immune reconstruction, we included patients who received first-line cART for at least 2 years and maintained persistent virological suppression (undetectable viral load) during the treatment. The immune reconstruction was defined as two successive CD4^+^ T-cell counts of more than 500 cells/μl after cART initiation, while poor immune reconstruction was defined as CD4^+^ T-cell count <500 cells/μl at all follow-up visits (Kelley et al., 2009; Li et al., 2022). In our study, we enrolled the newly diagnosed HIV-1-infected patients during 2015–2019; thus, there were two different criteria for these patients of whether receiving cART in China. For patients with HIV-1 diagnosed in 2015, only patients with CD4^+^ T-cell count <500 cells/μl were suggested to receive the cART. However, in June 2016, China officially issued the test-and-immediately-treat policy, which meant that all HIV-infected patients were suggested to receive immediate antiretroviral therapy upon an HIV diagnosis. Since then, patients who were willing to receive treatment could immediately initiate the cART when they were diagnosed with HIV infection. The mean time between HIV-1 diagnosis and cART initiation was 206.60 days for all patients involved in our study from 2015 to 2019.

### Statistical Analyses

The difference in demographic information among epidemic branches was compared using the chi-square test or Fisher's exact probability test and Kruskal–Wallis test. The multivariate binomial logistic regression analysis was applied to identify the factors that influence the formation of the transmission network.

The Kruskal–Wallis test was conducted to compare the CD4^+^ T-cell count and plasma HIV-1 viral load among patients with different subtypes. The generalized additive mixed model (GAMM) was performed to estimate the annual decline rate of the CD4^+^ T-cell count. The Kaplan–Meier analysis was adopted to compare the progression of achieving immune reconstruction since the cART initiation among different subtypes. The primary end event was reaching immune reconstruction. The multivariate Cox proportional hazard model was used to further determine the impact of CRF01_AE/B recombinants on immune reconstruction. All statistical analyses were carried out using the R software (version 4.0.4).

## Results

### Evolutionary Analysis and Demographic Characteristics of CRF01_AE/B Recombinants

**Figure 1:**
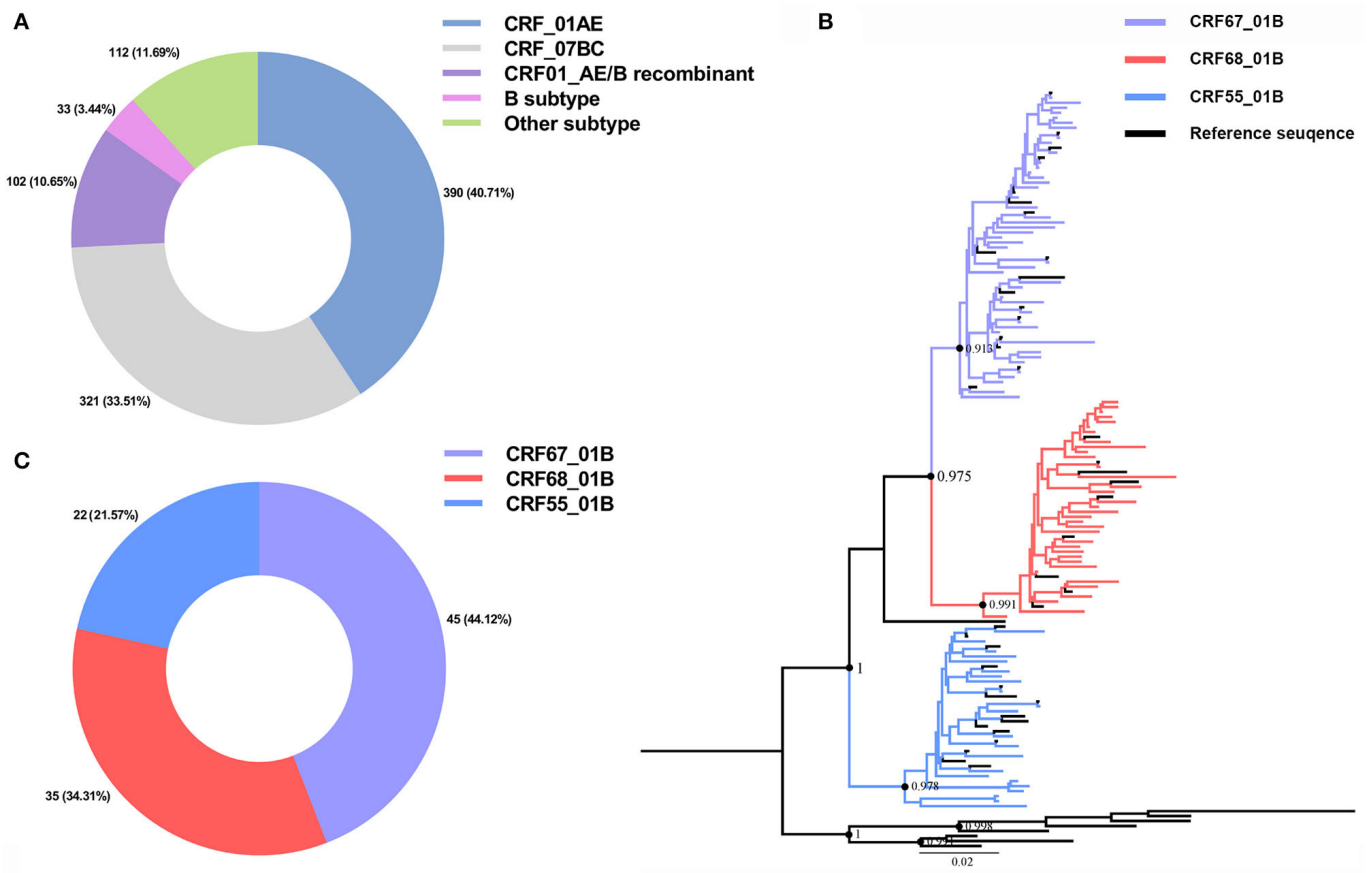
Human immunodeficiency virus 1 (HIV-1) sub-typing based on the 958 partial Pol region sequences from newly diagnosed patients in Nanjing during 2015–2019 and reference sequences. **(A)** The HIV-1 subtypes distribution in Nanjing during 2015–2019. **(B)** The phylogenetic tree analysis of 102 CRF01_AE/B recombinants sequences of Nanjing. The different colors represented different CRF01_AE/B recombinants sequences and reference sequences. **(C)** The distribution of three subtypes of CRF01_AE/B recombinants in Nanjing during 2015–2019.

We have collected the plasma samples from 1,013 HIV-1-infected patients in Nanjing from 2015 to 2019, among which we have successfully amplified 958 partial Pol sequences with an amplification rate of 94.57% (958/1,013). The subtypes of CRF_01AE (390/958, 40.71%) and CRF_07BC (321/958, 33.51%) were the most circulating strains in Nanjing (Figure 1A). A total of 102 CRF01_AE/B recombinants (102/958, 10.65%) have been identified including CRF67_01B (45/102, 44.12%), CRF68_01B (35/102, 34.31%), and CRF55_01B (22/102, 12.57%; Figures 1B,C). The demographic characteristics of CRF01_AE/B recombinants were shown in Table 1. We found that the majority of patients with CRF01_AE/B recombinants were men and infected with HIV-1 through men who have sex with men (MSM). The CRF01_AE/B recombinant profiles are shown in Supplementary Table 1.

**Table 1:** Demographic and clinical characteristics among three CRF01_AE/B recombinants.

**Variable**	**Number (%)/** **Median (IQR)**	**HIV-1 subtype**			***P-*** **value**
		**CRF67_01B**	**CRF68_01B**	**CRF55_01B**	
Sample size	102 (100.00%)	45 (44.12%)	35 (34.31%)	22 (21.57%)	—
Age	28.00 (22.00–38.25)	29.00 (23.00–40.50)	28.00 (22.00–40.00)	24.00 (20.00–29.00)	0.066
Gender					
Male	101 (99.02%)	45 (100.00%)	34 (97.14%)	22 (100.00%)	
Female	1 (0.98%)	0 (0.00%)	1 (2.86%)	0 (0.00%)	
Occupation					**0.001**
Student	29 (28.43%)	8 (17.78%)	8 (22.86%)	13 (59.09%)	
Non-student	73 (71.57%)	37 (82.22%)	27 (77.14%)	9 (40.91%)	
Education background					0.157
Technical secondary school or below	34 (33.33%)	15 (33.33%)	15 (42.86%)	4 (18.18%)	
Junior college or above	68 (66.67%)	30 (66.67%)	20 (57.14%)	18 (81.82%)	
Marital status					0.062
Spinsterhood	67 (65.69%)	28 (62.22%)	20 (57.14%)	19 (86.36%)	
Married	35 (34.31%)	17 (37.78%)	15 (42.86%)	3 (13.64%)	
Sexual orientation					0.341
Homosexuality	41 (40.20%)	19 (42.22%)	16 (45.71%)	6 (27.27%)	
Heterosexuality	61 (59.80%)	26 (57.78%)	19 (54.29%)	16 (72.73%)	
Infection route					0.403
MSM	89 (87.25%)	40 (86.95%)	28 (82.35%)	21 (95.45%)	
HET	13 (12.75%)	6 (13.04%)	6 (17.65%)	1 (4.55%)	
Number of sex mate					0.217
≤ 5	72 (70.59%)	28 (62.22%)	28 (80.00%)	16 (72.73%)	
>5	30 (29.41%)	17 (37.78%)	7 (20.00%)	6 (27.27%)	
STD history					0.259
No	63 (61.76%)	24 (53.33%)	23 (65.71%)	16 (72.73%)	
Yes	39 (38.24%)	21 (46.67%)	12 (34.29%)	6 (27.27%)	
Condom use					0.906
Regular use	47 (46.08%)	22 (48.89%)	15 (42.86%)	10 (45.45%)	
Occasional or never use	55 (53.92%)	24 (53.33%)	20 (57.14%)	12 (54.55%)	
Casual sexual behavior					**0.009**
No	26 (25.49%)	7 (15.56%)	16 (45.71%)	9 (40.91%)	
Yes	76 (74.51%)	38 (84.44%)	19 (54.29%)	13 (59.09%)	
Regular sexual behavior					0.373
No	43 (42.16%)	24 (53.33%)	19 (54.29%)	8 (36.36%)	
Yes	59 (57.84%)	21 (46.67%)	16 (45.71%)	14 (63.64%)	
Baseline CD4^+^ T cell count at HIV-1 infection diagnosis	384.50 (223.75–535.25)	343.00 (224.00–463.75)	424.00 (151.00–637.00)	459.00 (303.00–547.00)	0.207
Baseline CD4^+^ T cell count at cART initiation	297.00 (65.00–413.00)	294.00 (58.00–378.00)	212.50 (25.00–400.25)	397.50 (286.50–516.25)	**0.033**
Baseline viral load at cART initiation	80,800 (25,400–142,000)	85,500 (22,850–173,750)	93,950 (30,400–103,000)	39,700 (22,600–93,050)	0.557
Age of cART initiation	29.00 (23.00–39.50)	29.00 (26.00–40.75)	32.00 (23.00–42.00)	24.50 (21.50–29.50)	0.084
cART regimen					0.287
EFV+TDF+3TC	63 (77.78%)	25 (69.44%)	24 (88.90%)	14 (77.78%)	
EFV+AZT+3TC	12 (14.82%)	8 (22.22%)	1 (3.70%)	3 (16.66%)	
NVP+AZT+3TC	2 (2.47%)	1 (2.78%)	1 (3.70%)	0 (0%)	
NVP+TDF+3TC	1 (1.23%)	1 (2.78%)	0 (0%)	0 (0%)	
DTG+TDF+3TC	2 (2.47%)	0 (0%)	1 (3.70%)	1 (5.56%)	
DTG+AZT+3TC	1 (1.23%)	1 (2.78%)	0 (0%)	0 (0%)	

*The bold values represent that the comparison between groups reaches statistical significance, namely P value less than 0.05*.

We also compared the demographic characteristics among three subtypes of CRF01_AE/B recombinants. There were statistical differences in the distribution of occupation (*P* = 0.001) and casual behavior (*P* = 0.009) and baseline CD4^+^ T-cell count at the cART initiation (*P* = 0.033) among these three subtypes (Table 1). Most CRF55_01B patients were students (13/22, 59.09%), whereas non-students accounted for the majority of subtypes of CRF67_01B (37/45, 82.22%) and CRF68_01B (27/35, 77.14%). Furthermore, the proportion of casual behavior in patients with CRF67_01B was much higher than the other two subtypes (i.e., CRF67_01B: 84.44%; CRF55_01B: 59.09%; and CRF68_01B: 54.29%). The patients with CRF55_01B had a higher baseline CD4^+^ T-cell count at cART initiation than other two subtypes (median CRF55_01B: 397.50; median CRF67_01B: 294.00; and median CRF68_01B: 212.50).

### Bayesian System Evolution Inference Analysis of CRF01_AE/B Recombinants

**Figure 2:**
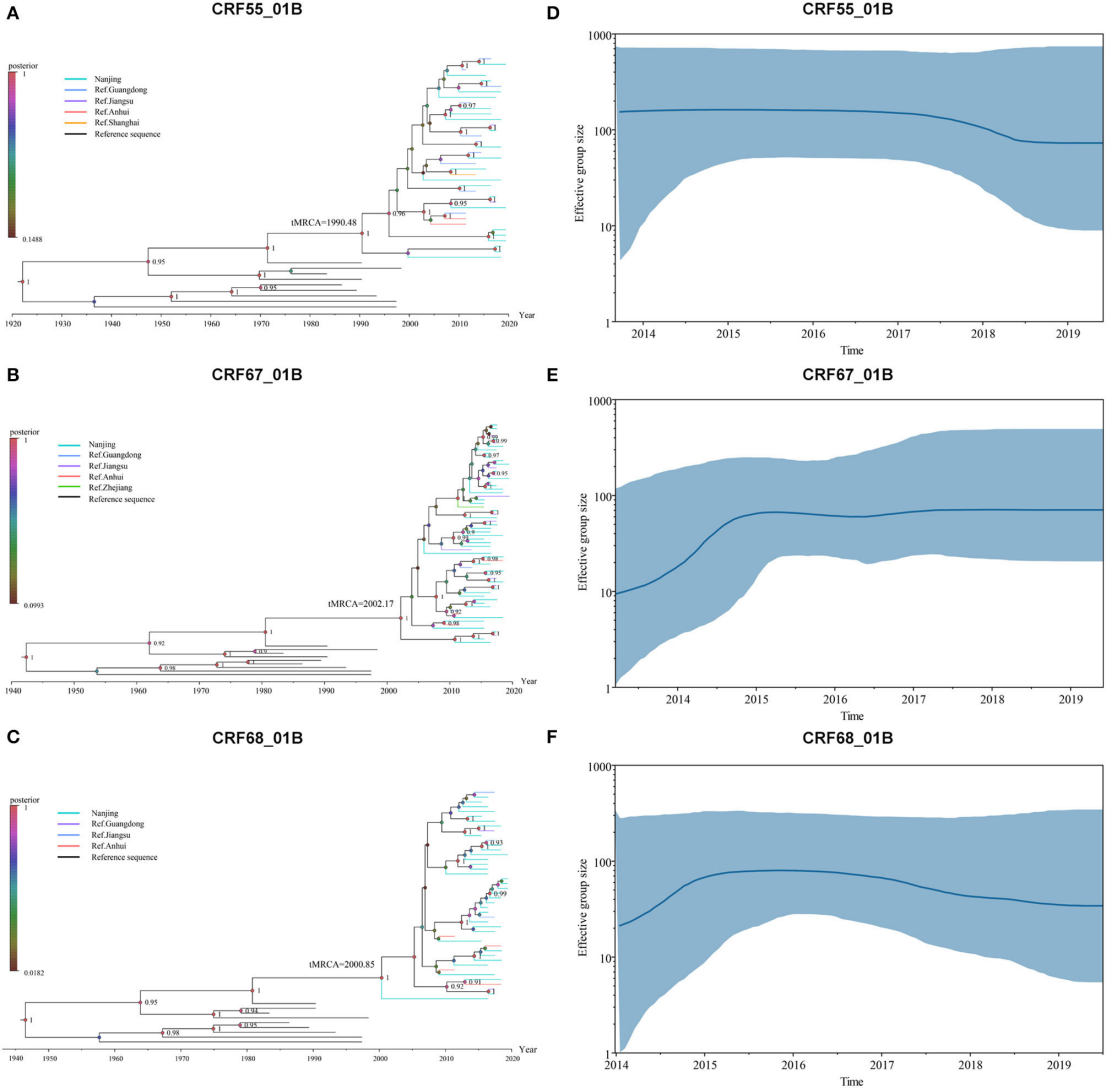
Bayesian system evolution analysis of CRF01_AE/B recombinants. **(A–C)** The time to most recent common ancestor (tMRCA) of CRF55_01B **(A)**, CRF67_01B **(B)**, and CRF68_01B **(C)** inferred by Bayesian evolution analysis. **(D–F)** Bayesian skyline plots for CRF55_01B **(D)**, CRF67_01B **(E)**, and CRF68_01B **(F)** in Nanjing. The line represented the effective sample size, and the shaded region depicted the upper and lower 95% highest posterior density interval (HPD) estimates.

The evolution rate and 95% highest posterior density (HPD) of CRF55_01B, CRF67_01B, and CRF68_01B were: 1.78 × 10^−2^ (95%HPD: 0.89 × 10^−2^-2.76 × 10^−2^) substitutions/site/year, 2.46 × 10^−2^ (95%HPD: 1.24 × 10^−2^-3.66 × 10^−2^) substitutions/site/year, and 2.04 × 10^−2^ (95%HPD: 0.87 × 10^−2^-3.31 × 10^−2^) substitutions/site/year, respectively. As shown in Figures 2A–C, the tMRC and 95%HPD of these three subtypes were CRF55_01B (1,990.48, 95%HPD: 1,976.81–1,997.57), CRF67_01B (2,002.179, 95%HPD: 1,993.10–2,004.81), and CRF68_0,1B (2,000.35, 95%HPD: 1,989.79–2,006.26). The epidemic trend of three subtypes was shown in the BSP. We observed that CRF55_01B in Nanjing had a rapid decline stage during 2017–2019, while the CRF67_01B and CRF68_01B have experienced an initial phase of fast growth during 2014–2015 and then remained stable (Figures 2D–F).

### Transmission Network Analyses

**Figure 3:**
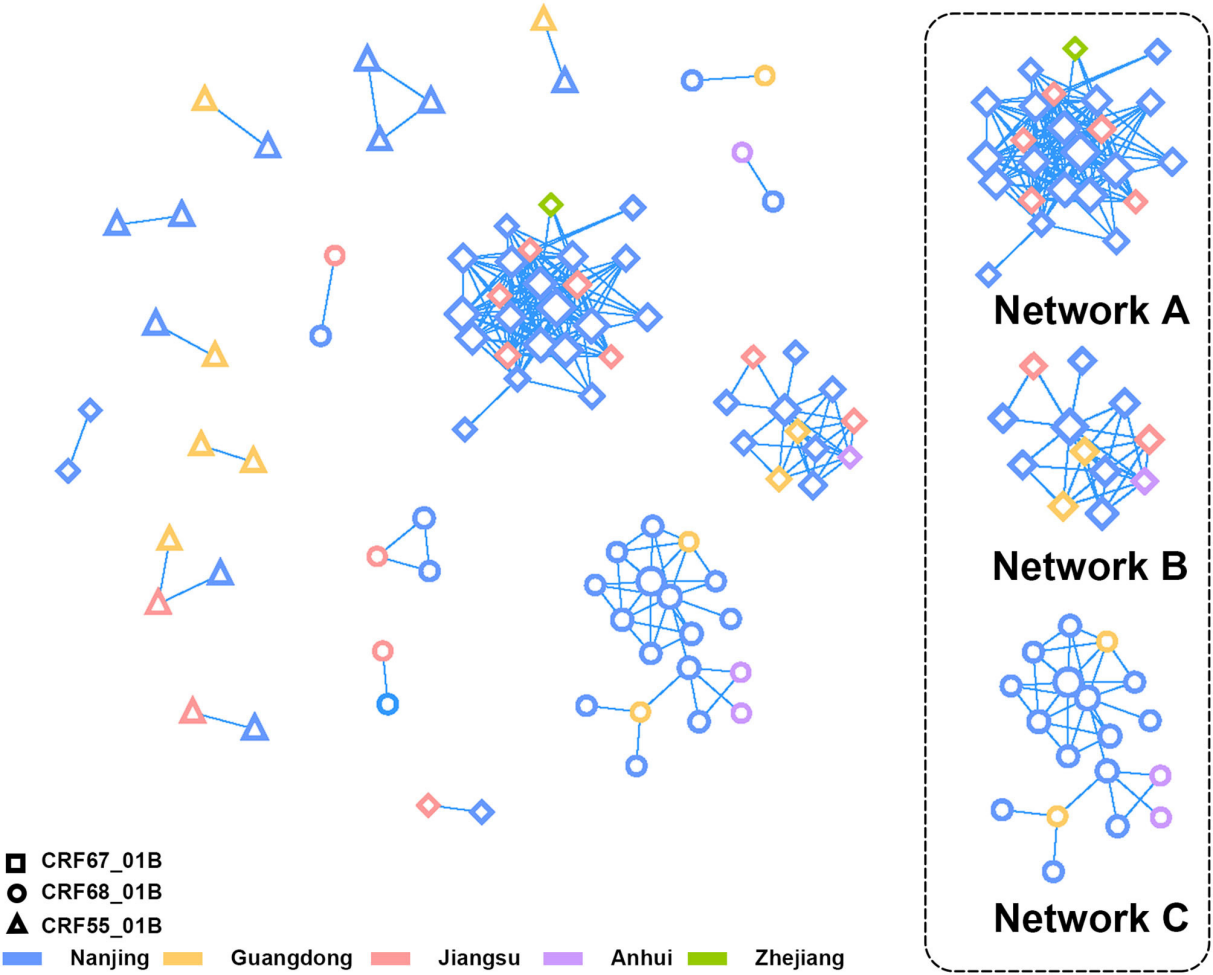
The transmission network analysis of CRF01_AE/B recombinants in Nanjing. The different shapes and colors of nodes represented different CRF01_AE/B recombinants and the district of Pol sequences profiles, respectively. The three lager clusters on the right side were composed of CRF67_01B (Network A and B) and CRF68_01B (Network C).

At the criterion of pairwise genetic distance ≤1.0%, we constructed the transmission network of CRF01_AE/B recombinants, which contained 59 sequences from Nanjing and 27 reference sequences (Figure 3). The network access rate of the CRF01_AE/B recombinants sequence in Nanjing was 57.84% (59/102). The sequences in network A and B were CRF67_01B strain, and the network C was consisted of CRF68_01B strain. The reference sequence in these three networks came from Jiangsu, Zhejiang Guangdong, and Anhui provinces. The CRF55_01B virus was included mostly in small transmission clusters, and the transmission within cases was not closely connected.

When comparing the demographic characteristics between patients within and outside the network, we discovered the differences in distribution of CRF01_AE/B recombinants, occupation, infection route, and history of sexually transmitted disease (STD) at the threshold of *P* < 0.1 (Supplementary Table 2). Over half of the sequences included in the transmission network were of CRF67_01B strain (32/59, 54.24%). Most of the patients in the network were non-students (48/59, 81.36%), and the main infection occurred through MSM (55/59, 93.22%). In addition, a much higher proportion of patients in the network have had a history of STD than patients outside the network. The multivariate logistic regression analysis revealed that occupation (non-students vs. students, *P* = 0.015, odds ratio (OR) = 3.331, 95%CI = 1.266–8.768), infection route (MSM vs. heterosexual contact, *P* = 0.021, OR = 5.230, 95%CI = 1.279–21.391), and history of STD (yes vs. no, *P* = 0.007, OR = 3.696, 95%CI = 1.418–9.632) were the main factors that affect the network formation (Supplementary Table 3).

### The Impact of CRF01_AE/B Recombinants on CD4^+^ T-Cell Count Change Before cART

**Figure 4:**
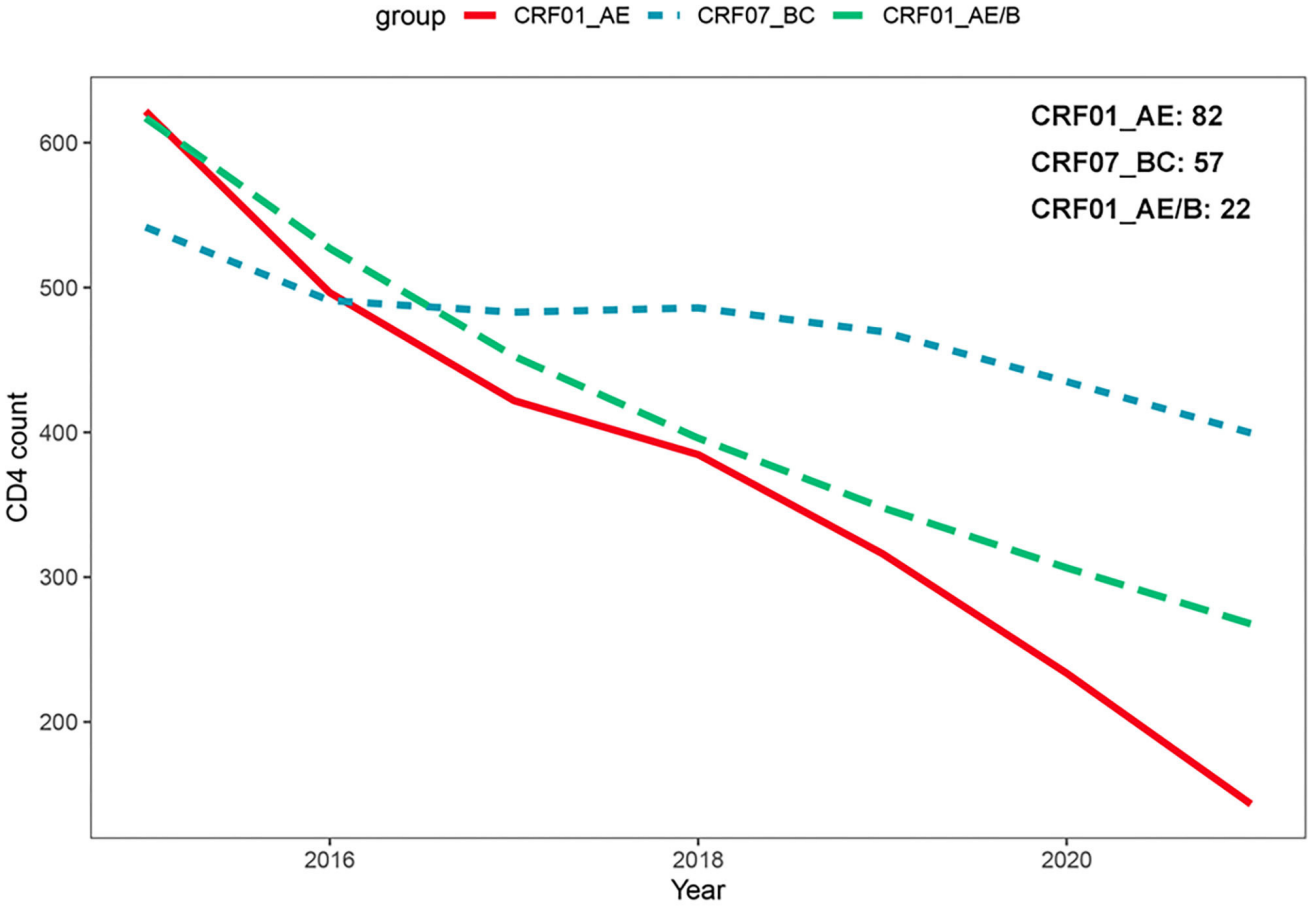
Comparison of CD4^+^ T-cell count change over time among different HIV-1 recombinant profiles based on generalized additive mixed model. The decline rate of CD4^+^ T-cell count in patients with CRF01_AE/B recombinants was significantly faster than CRF07_BC patients (interaction *P* < 0.01) but not different from CRF01_AE patients (interaction *P* = 0.63).

We attempted to explore the difference of CD4^+^ T-cell count declining rates among three subtypes. A total of 161 patients with at least two CD4^+^ T-cell count records in different years were chosen for the analysis, including CRF_01AE (*n* = 82), CRF07_BC (*n* = 57), and CRF01_AE/B recombinants (*n* = 22; Supplementary Figure 1). As displayed in Supplementary Table 4, there were no differences in the clinical and demographic characteristics reported to affect CD4^+^ T-cell count change among three subtypes, i.e., baseline CD4^+^T count and age at HIV-1 diagnosis, diagnosis time, and infection route (Wei et al., 2021; James and Dixit, 2022). We operated the square root transformation of CD4^+^ T-cell count to linearize the variation trend. We observed that patients with all these three subtypes showed the annually decrease trend of CD4^+^ T-cell count (CRF01_AE, β: −1.99 (cell/μl)^1/2^/year, 95% CI: −2.34 to −1.63; CRF07_BC, β: −0.54 (cell/μl)^1/2^/year, 95% CI: −0.86 to −0.21; CRF01_AE/B recombinants, β: −1.66 (cell/μl)^1/2^/year, 95% CI: −2.42 to −0.90; Table 2). However, the change tendency of CD4 ^+^ T-cell count differed among three subtypes. The decline rate of CD4^+^ T cell count in patients with CRF01_AE/B recombinants was significantly faster than in patients with CRF07_BC (interaction *P* < 0.01) but not different from patients with CRF01_AE (interaction *P* = 0.63; Table 2 and Figure 4).

**Table 2:** The analysis of annually decline rate of CD4^+^ T-cell count using generalized additive mixed model (GAMM).

**Variable**	**Decline rate (cell/μl)^½^/year**		
	**β**	**95%CI**	***P-*** **value**
CRF01_AE	−1.99	−2.34 ~−1.63	**<0.01**
CRF07_BC	−0.54	−0.86 ~−0.21	**<0.01**
CRF01_AE/B recombinant	−1.66	−2.42 ~−0.90	**<0.01**
Reference **(**CRF01_AE/B recombinant)	—	—	—
Year^*^ CRF01_AE (test for interaction)	−0.16	−0.83 ~ 0.50	0.63^a^
Year^*^ CRF07_BC (test for interaction)	1.20	0.53 ~ 1.86	**<0.01** ^a^

a *P-value was adjusted by CD4^+^ T cell count at HIV-1 diagnosis, age at HIV-1 diagnosis, infection route and STD history*.

*The bold values represent that the comparison between groups reaches statistical significance, namely P value less than 0.05*.

### The Impact of CRF01_AE/B Recombinants on Immune Reconstruction After cART

A total of 329 patients with sustained viral suppression during cART were chosen in the following analysis, including 159 CRF01_AE, 132 CRF07_BC, and 38 CRF01_AE/B recombinants patients (Supplementary Figure 1).

**Figure 5:**
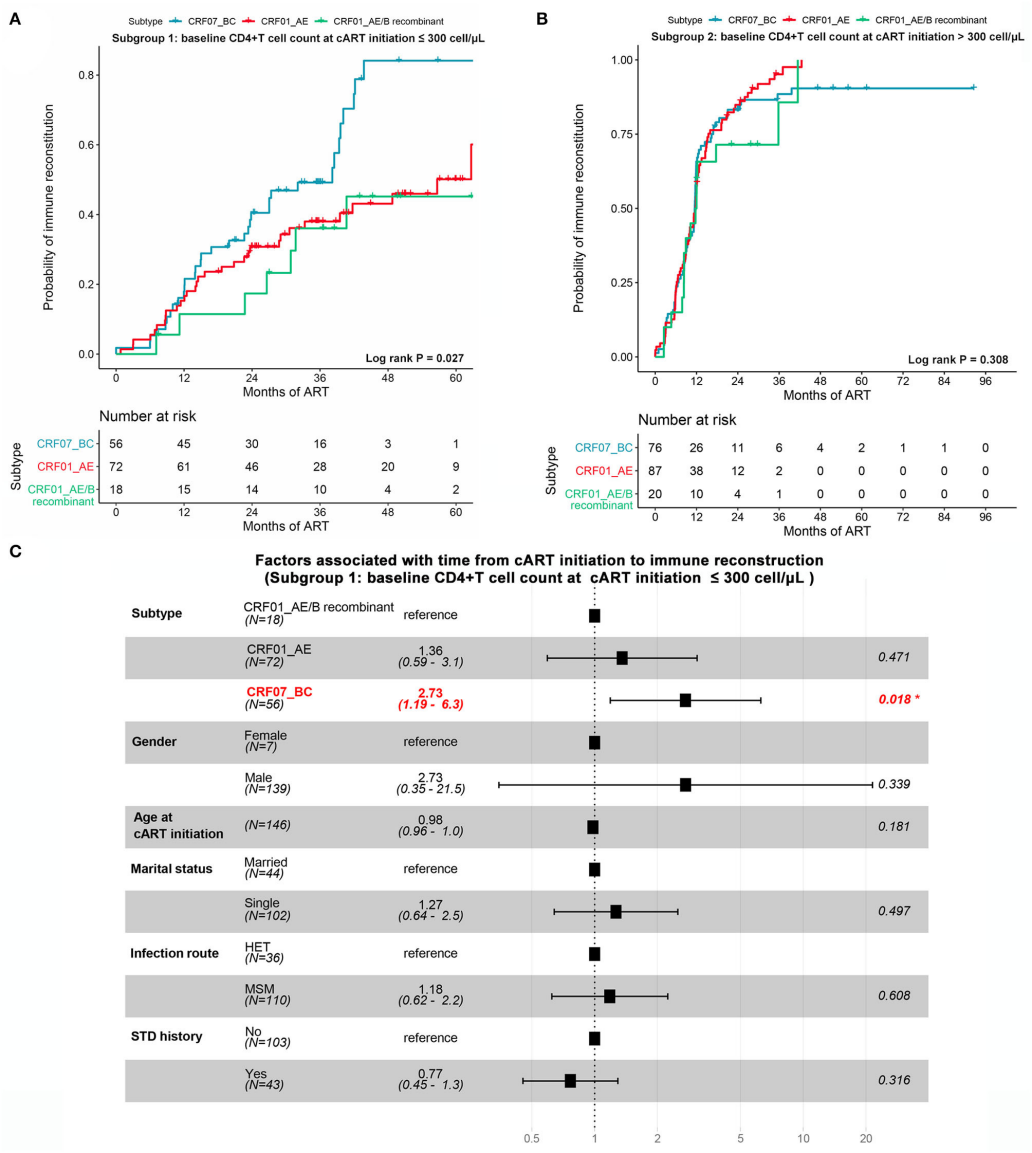
The impact of CRF01_AE/B recombinants on immune reconstruction after cART. **(A,B)** The possibility of achieving immune reconstruction (from cART initiation to CD4^+^ cell count >500 cells/μl) among different HIV-1 subtypes stratified by baseline CD4^+^ T-cell count at cART initiation. **(A)** Patients with CD4^+^ T-cell count at cART initiation ≤300 cells/μl. **(B)** Patients with CD4^+^ T-cell count at cART initiation >300 cells/μl. The statistical difference was examined using the log-rank test. **(C)** The forest plot showing the factors associated with immune reconstruction at subgroup of patients with CD4^+^ T-cell count ≤300 cells/μl. Patients with CRF01_AE/B recombinants were less likely to reach a normal CD4^+^ T-cell count than CRF07_BC patients but not different from CRF_01AE patients.

Due to the important role of baseline CD4^+^ T-cell count at the cART initiation in immune recovery, we assessed the progression of immune reconstruction in two subgroups (i.e., subgroup 1 ≤300 cells/μl and subgroup 2 >300 cells/μl). In subgroup 1, we found that the probability of achieving immune reconstruction in CRF01_AE/B recombinants was significantly lower than CRF07_BC but similar to CRF01_AE (log rank *P* = 0.027; Figure 5A). However, in subgroup 2, no difference was observed among three subtypes (log rank *P* = 0.308; Figure 5B). Furthermore, after adjusting the baseline characteristic, the Cox regression analysis suggested that the probability of achieving immune reconstruction in CRF01_AE/B recombinants was significantly lower than CRF07_BC in subgroup1 but did not differ from CRF_01AE (Figure 5C). We also compared the immune reconstruction between different HIV-1 subtypes in the whole patients (both subgroup1 and subgroup2). As shown in Supplementary Figure 2A, CRF01_AE/B recombinants patients showed slower progression of immune reconstruction than CRF07_BC (*P* = 0.054). However, the multivariate Cox regression analysis suggested that the baseline CD4^+^ T-cell count at the cART initiation was the main factor that exerted a great impact on CD4^+^ T-cell recovery instead of HIV-1 subtypes (Supplementary Figure 2B).

In summary, these data suggested that the impact of baseline CD4^+^ T-cell count at cART initiation on immune reconstitution might be greater than HIV-1 subtypes. However, for patients with lower baseline CD4^+^ T-cell count at the cART initiation, CRF01_AE/B recombinants might have unfavorable influence on CD4^+^ T-cell recovery.

## Discussion

In this study, we systematically explored the molecular epidemiological and immunological characteristics of CRF01_AE/B recombinants in Nanjing for the first time. Unlike other cities of Eastern China, in Nanjing, CRF67_01B and CRF68_01B were the main circulating strains of CRF01_AE/B recombinants instead of CRF55_01B. CRF01_AE/B recombinants patients showed faster decline rates of CD4^+^ T-cell count and slower immune reconstruction progression than CRF07_BC but similar to CRF01_AE.

The CRF55_01B was the major subtype of CRF01_AE/B recombinants in China. It has been first identified among MSM in Shenzhen and has now spread throughout most Southern and Eastern provinces of China, with the prevalence ranging from 1.5 to 12.5% (Lan et al., 2018). Remarkably, the CRF55_01B has overtaken subtype B as the third predominant strain in Shenzhen since 2012 (Wei et al., 2021). However, unlike the epidemic situation of CRF01_AE/B recombinants in other cities of Southern and Eastern provinces (Han et al., 2015; Zai et al., 2020), CRF67_01B and CRF68_01B were more prevalent than CRF55_01B in Nanjing. In addition, CRF55_01B has gone through a rapid decline since 2017, but CRF67_01B and CRF68_01B have undergone a phase of fast growth during 2014–2015, after which CRF67_01B remained stable while CRF68_01B declined slightly. Similar to our results, the higher proportion of CRF67_01B than CRF55_01B was also reported in Jiangsu Province from previous studies (Yang et al., 2018; Yin et al., 2021). When comparing the demographic characteristics among different subtypes, we found that patients with CRF67_01B and CRF68_01B were mostly non-student population, whereas over half of the CRF55_01B patients were student population. The main infection route of CRF67_01B and CRF68_01B was MSM as same as CRF55_01B. However, the occurrence of casual sexual behavior was more frequent in patients with CRF67_01B than that with CRF68_01B or CRF55_01B, which might partly explain the higher prevalence and steady growth trend of CRF67_01B strain in Nanjing.

The transmission network analysis revealed three main transmission clusters of the CRF01_AE/B recombinants in Nanjing, which were composed of CRF67_01B and CRF68_01B. These three clusters showed the potential transmission relationship between Nanjing and other cities of Jiangsu, Anhui, Zhejiang, and Guangdong provinces. It should be on the alert for the spread of CRF67_01B and CRF68_01B from Nanjing to other provinces around Jiangsu (Anhui and Zhejiang), although the dominant epidemic CRF01_AE/B recombinants remained CRF55_01B in these surrounding provinces (Zhang et al., 2017, 2020; Zheng et al., 2021). As reported previously, the Beijing–Kowloon railways might account for the potential transmission relationship between Nanjing and Guangdong provinces (Gan et al., 2021). CRF55_01B in Nanjing mostly formed smaller transmission clusters with relatively loose transmission correlation between cases, which is consistent with the Zheng et al. research (Zheng et al., 2020). The small transmission cluster of CRF55_01B might be attributed to insufficient sampling and lack of linkages. It still required further extensive sampling to reveal the existence of additional CRF55_01B clusters in Nanjing.

We also described the immunological characteristics of CRF01_AE/B recombinants in this study. CRF01_AE/B recombinants manifested similar negative effects on disease progression and immune reconstitution as CRF01_AE, more severe than CRF07_BC. Notably, the CRF_01AE subtype has been reported to be more pathogenic than other subtypes in the studies of Asian population, such as lower level of baseline CD4^+^ T-cell count, greater CD4^+^ T-cell count loss, shorter median survival, and poorer immune reconstruction ability, compared with other subtypes (Nelson et al., 2007; Rangsin et al., 2007; Ng et al., 2011; Li X. et al., 2014; Li et al., 2022). The pathogenic impact of CRF01_AE on HIV-1 disease progression might be explained by the larger proportion of CXCR4-tropic viruses and higher frequency of R5 to X4/DM switch (Li X. et al., 2014; Li Y. et al., 2014; Cui et al., 2019; Ge et al., 2021). In general, our study suggested that consistent with CRF01_AE, infection of CRF01_AE/B recombinants might accelerate progression to AIDS. Meanwhile, the virus tropism in CRF01_AE/B recombinants needed further experimental investigation.

There were several limitations in this study. First, this study only enrolled the newly diagnosed patients, and some patients with direct transmission relationship might be missed in the process of transmission network construction due to non-new diagnosis or sampling error, causing missing links in the transmission chain, such as relatively loose clusters of CRF55_01B. Furthermore, these networks inferred from viral sequences did not directly establish complete epidemiological links. The real-world epidemiological survey was required for further verification. Second, although the patients included in our study were newly diagnosed, the date of seroconversion in these patients was lacking, a common limitation in studies about HIV disease progression (Vasan et al., 2006). Therefore, the decline rate of the CD4^+^ T-cell count was calculated more from diagnosed time than from the time of infection. Nevertheless, there was no difference in the baseline CD4^+^ T-cell count at the time of infection diagnosed among different subtypes, which could partially control the influence of infection duration variability on the inference about disease progression. In addition, although we observed the difference in the baseline clinical information at HIV-1 diagnosis or at the cART initiation among three subtypes, we could not attribute the difference into different HIV-1 subtypes due to the lack of accurate infection time. Third, the presence of co-infection (such as TB, HCV, and HBV) and other opportunistic infections was also an important factors affecting immune reconstruction after cART. Further studies containing this clinical information were required to verify our results. Finally, we failed to separately explore the impact of different subtypes of CRF01_AE/B recombinants on immunological responses because of the limited sample size and incomplete clinical data. The heterogeneity across different subtypes of CRF01_AE/B recombinants might influence the strength of our results.

## Conclusion

In summary, CRF01_AE/B recombinants might become the new HIV-1 strains that would commonly transmit within MSM in Nanjing. The CRF67_01B and CRF68_01B were the main CRF01_AE/B recombinants in Nanjing and have formed large transmission clusters between Nanjing and other provinces. CRF01_AE/B recombinants were associated with a fast decline rate of CD4^+^ T-cell count and slow progression of immune reconstruction completion, implying the possibility of rapid disease progression and poor clinical outcome. It is necessary to strengthen the epidemiological surveillance of CRF01_AE/B recombinants in Nanjing.

## Data Availability Statement

The datasets presented in this study can be found in online repositories. The names of the repository/repositories and accession number(s) can be found in the article/Supplementary Material.

## Ethics Statement

The studies involving human participants were reviewed and approved by the Human Research Ethics Committee of Zhongda Hospital affiliated to Southeast University (No. 2017ZKKYSB045). The patients/participants provided their written informed consent to participate in this study.

## Author Contributions

YG, WL, and PW conceived and designed the study. YL, XL, GFu, and GD collected the samples and demographic data. JL, GFe, HL, and ZW collected the clinical data. YG and YL peformed the experiments and analyzed the results. YG completed the writing of the manuscript. WL and PW reviewed and edited the manuscript. All authors contributed to the article and approved the submitted version.

## Funding

This study was supported by National Science and Technology Major Projects (2018ZX10715-002), Humanities and Social Sciences of Ministry of Education Planning Fund of China (No. 16YJA84001), and Youth Program of National Natural Science Foundation of China (82103896).

## Conflict of Interest

The authors declare that the research was conducted in the absence of any commercial or financial relationships that could be construed as a potential conflict of interest.

## Publisher's Note

All claims expressed in this article are solely those of the authors and do not necessarily represent those of their affiliated organizations, or those of the publisher, the editors and the reviewers. Any product that may be evaluated in this article, or claim that may be made by its manufacturer, is not guaranteed or endorsed by the publisher.
