# Supplementary material for: The Molecular Epidemiological and Immunological Characteristics of HIV-1 CRF01_AE/B Recombinants in Nanjing, China
Source: Front Microbiol. 2022 Jul 15;13:936502. doi: 10.3389/fmicb.2022.936502 (PMC9335199; doi:10.3389/fmicb.2022.936502)
Supplement: Supplementary file 2 [file Table_2.DOCX]

**Table S2. Epidemiological characteristics of patients within and outside network**

| Variable | | CRF01_AE/B recombinant patients | | *P* value |
| --- | --- | --- | --- | --- |
|  |  | Within network | Outside network |  |
| Subtype | |  |  | **0.057** |
|  | CRF67_01B | 32 (54.24%) | 13 (30.23%) |  |
|  | CRF68_01B | 17 (28.81%) | 18 (41.86%) |  |
|  | CRF55_01B | 10 (16.95%) | 12 (27.91%) |  |
| Age | | 28.00 (23.00-39.00) | 26 (22.00-35.00) | 0.517 |
| Gender | |  |  | 1.000^a^ |
|  | Male | 58 (98.31%) | 43 (100%) |  |
|  | Female | 1 (1.69%) | 0 (0%) |  |
| Occupation | |  |  | **0.010** |
|  | Student | 11 (18.64%) | 18 (41.86%) |  |
|  | Non-student | 48 (81.36%) | 25 (58.14%) |  |
| Education background | |  |  | 0.777 |
|  | Technical secondary school or below | 19 (32.2%) | 15 (34.88%) |  |
|  | Junior college or above | 40 (67.8%) | 28 (65.12%) |  |
| Marital status | |  |  | 0.918 |
|  | Spinsterhood | 39 (66.10%) | 28 (65.12%) |  |
|  | Married | 20 (33.90%) | 15 (34.88%) |  |
| Infection route | |  |  | **0.034** |
|  | MSM | 55 (93.22%) | 34 (79.07%) |  |
|  | HET | 4 (6.78%) | 9 (20.93%) |  |
| Number of sex mate | |  |  | 0.776 |
|  | ≤5 | 41 (69.49%) | 31 (72.09%) |  |
|  | >5 | 18 (30.51%) | 12 (27.91%) |  |
| STD history | |  |  | **0.002** |
|  | No | 29 (49.15%) | 34 (79.07%) |  |
|  | Yes | 30 (50.85%) | 9 (20.93%) |  |
| Condom use | |  |  | 0.466 |
|  | Regular use | 29 (49.15%) | 18 (41.86%) |  |
|  | Occasional or never use | 30 (50.85%) | 25 (58.14%) |  |
| Casual sexual behavior | |  |  | 0.278 |
|  | No | 16 (27.12%) | 16 (37.21%) |  |
|  | Yes | 43 (72.88%) | 27 (62.79%) |  |
| Regular sexual behavior | |  |  | 0.841 |
|  | No | 30 (50.85%) | 21 (48.84%) |  |
|  | Yes | 29 (49.15%) | 22 (51.16%) |  |

^a^ *P* value obtained by Fisher's Exact Test
