# Supplementary material for: The Molecular Epidemiological and Immunological Characteristics of HIV-1 CRF01_AE/B Recombinants in Nanjing, China
Source: Front Microbiol. 2022 Jul 15;13:936502. doi: 10.3389/fmicb.2022.936502 (PMC9335199; doi:10.3389/fmicb.2022.936502)
Supplement: Supplementary file 3 [file Table_3.DOCX]

**Table S3. Multivariate logistic regression analysis of factors related to network formation**

| Variable | Classification | *β* value | S.E. | *Waldχ*^2^ value | *P* value | *OR* | 95%*CI* |
| --- | --- | --- | --- | --- | --- | --- | --- |
| Occupation | Student |  |  |  |  |  |  |
|  | Non-student | 1.203 | 0.494 | 5.939 | 0.015 | 3.331 | 1.266-8.768 |
| Infection route | Heterosexual contact |  |  |  |  |  |  |
|  | MSM | 1.654 | 0.719 | 5.299 | 0.021 | 5.230 | 1.279-21.391 |
| STD history | No |  |  |  |  |  |  |
|  | Yes | 1.307 | 0.489 | 7.153 | 0.007 | 3.696 | 1.418-9.632 |
