# Supplementary material for: The Molecular Epidemiological and Immunological Characteristics of HIV-1 CRF01_AE/B Recombinants in Nanjing, China
Source: Front Microbiol. 2022 Jul 15;13:936502. doi: 10.3389/fmicb.2022.936502 (PMC9335199; doi:10.3389/fmicb.2022.936502)
Supplement: Supplementary file 4 [file Table_4.DOCX]

**Table S4. Comparison of clinical characteristics reported to affect CD4^+^ T cell count change among three subtypes**

| Variable | | CRF_01AE | CRF_07BC | CRF01_AE/B recombinants | *P* value |
| --- | --- | --- | --- | --- | --- |
| Baseline CD4^+^ T count at HIV-1 diagnosis | | 527.50  (433.75,669.25) | 554.50  (413.75,665.25) | 600.50  (448.50,718.50) | 0.697^a^ |
| Age at HIV-1 diagnosis | | 28.00  (24.00, 34.75) | 25.00  (21.00,31.00) | 28.50  (24.00, 33.75) | 0.069^a^ |
| Diagnosis time | |  |  |  |  |
|  | 2015 | 49(61.25%) | 34(60.71%) | 13(59.09%) | 0.711^b^ |
|  | 2016 | 8(10.00%) | 6(10.71%) | 5(22.72%) |  |
|  | 2017 | 11(13.75%) | 8(14.29%) | 1(4.55%) |  |
|  | 2018 | 9(11.25%) | 5(8.93%) | 1(4.55%) |  |
|  | 2019 | 3(3.75%) | 3(5.36%) | 2(9.09%) |  |
| Infection route | |  |  |  |  |
|  | MSM | 66(83.50%) | 43(76.79%) | 19(86.36%) | 0.638^b^ |
|  | HET | 14(17.50%) | 13(23.21%) | 3(13.64%) |  |

a: Kruskal Wallis test; b: Fisher’s exact test
