# Supplementary material for: The Molecular Epidemiological and Immunological Characteristics of HIV-1 CRF01_AE/B Recombinants in Nanjing, China
Source: Front Microbiol. 2022 Jul 15;13:936502. doi: 10.3389/fmicb.2022.936502 (PMC9335199; doi:10.3389/fmicb.2022.936502)

Figure S1. The flow chart of immunological status analysis. The flow chart described the sample size and selection process.

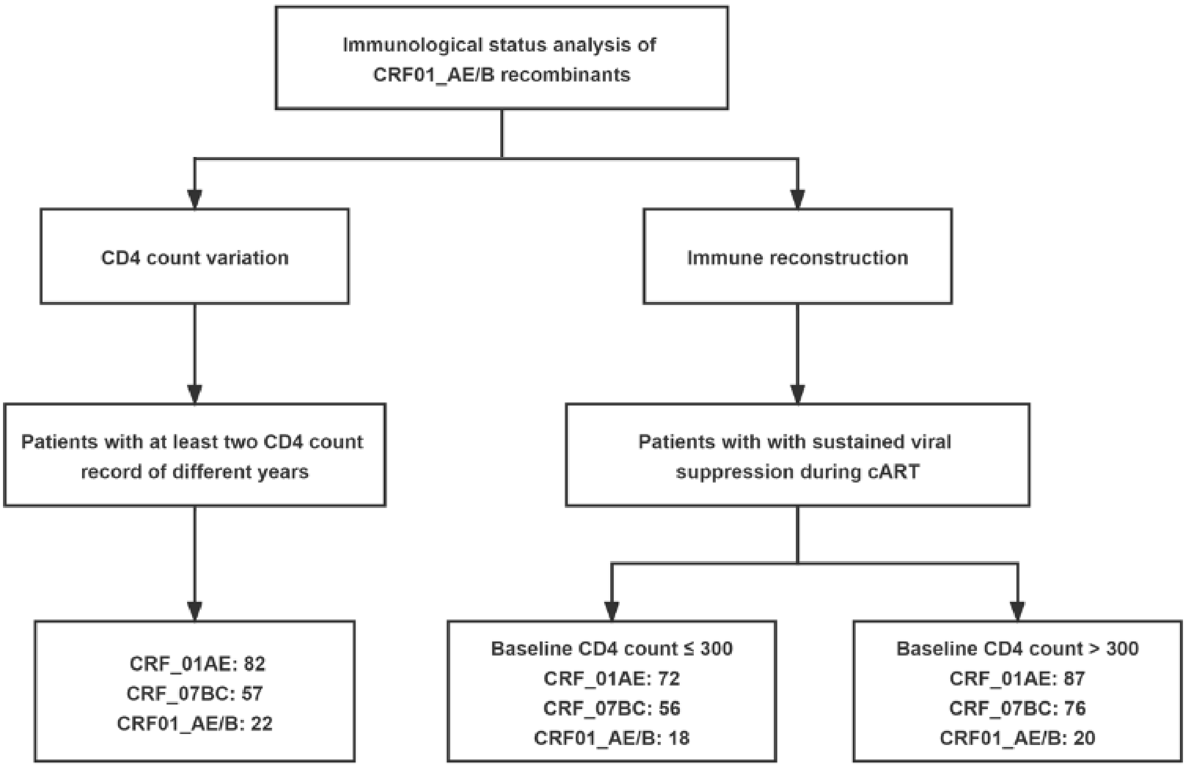

Supplement: Supplementary file 5 [file Data_Sheet_1.PDF]
