# Supplementary material for: The Molecular Epidemiological and Immunological Characteristics of HIV-1 CRF01_AE/B Recombinants in Nanjing, China
Source: Front Microbiol. 2022 Jul 15;13:936502. doi: 10.3389/fmicb.2022.936502 (PMC9335199; doi:10.3389/fmicb.2022.936502)

Figure S2. Comparison of immune reconstruction progression among three subtypes in the whole patients (both subgroup1 and subgroup2). (**A**) The possibility of achieving immune reconstruction among CRF01_AE, CRF_07BC and CRF01_AE/B recombinants. CRF01_AE/B recombinants showed slower progression of CD4+ T cell recovery than CRF07_BC (P = 0.054), although there was no statistical difference. The statistical difference was examined by Log-rank test. (**B**) The forest plot showing the factors associated with immune reconstruction in the whole patients. The baseline CD4^{+} T cell count at cART initiation was the main factor that exerted great impact on immune reconstruction.

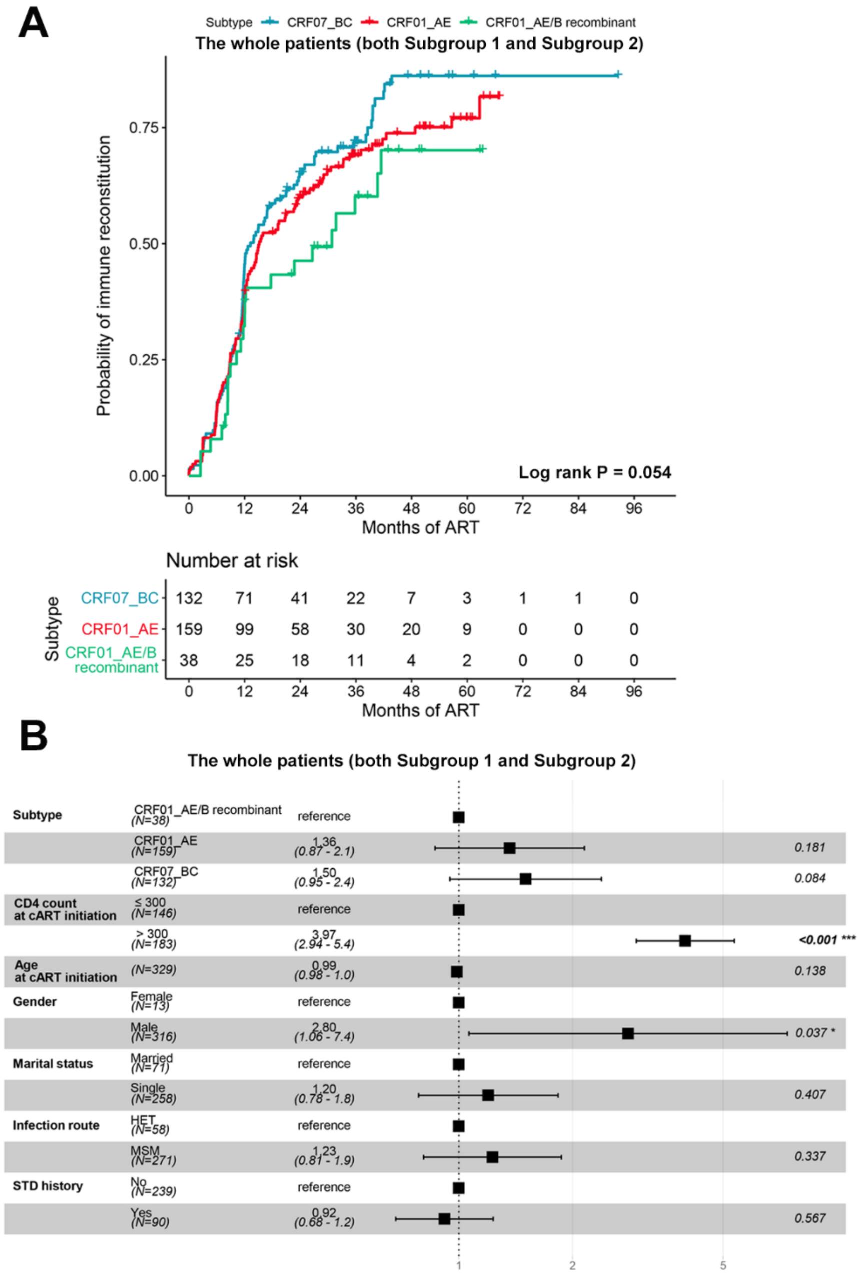

Supplement: Supplementary file 6 [file Data_Sheet_2.PDF]
